# Race development and performance-determining factors in a mass-start cross-country skiing competition

**DOI:** 10.3389/fspor.2022.1094254

**Published:** 2023-01-10

**Authors:** Trine M. Seeberg, Jan Kocbach, Hanna Wolf, Rune Kjøsen Talsnes, Øyvind B. Sandbakk

**Affiliations:** ^1^ Centre for Elite Sports Research, Department of Neuromedicine and Movement Science, Norwegian University of Science and Technology, Trondheim, Norway; ^2^ Smart Sensor and Microsensor System, SINTEF Digital, SINTEF AS, Oslo, Norway; ^3^ Department of Sports Science and Physical Education, Nord University, Bodø, Norway

**Keywords:** wearable sensors, GNSS - global navigation satellite system, skate, XC skiing, mass-start, cross-country skier

## Abstract

**Introduction:**

Although five of six Olympic events in cross-country skiing involve mass-starts, those events are sparsely examined scientifically. Therefore, in this study, we investigated speed profiles, pacing strategies, group dynamics and their performance-determining impact in a cross-country skiing mass-start competition.

**Methods:**

Continuous speed and position of 57 male skiers was measured in a six-lap, 21.8 km national mass-start competition in skating style and later followed up with an online questionnaire. Skiers ranked from 1 to 40 were split into four performance-groups: R1–10 for ranks 1 to 10, R11–20 for ranks 11 to 20, R21–30 for ranks 21 to 30, and R31–40 for ranks 31 to 40.

**Results:**

All skiers moved together in one large pack for 2.3 km, after which lower-performing skiers gradually lost the leader pack and formed small, dynamic packs. A considerable accordion effect occurred during the first half of the competition that lead to additional decelerations and accelerations and a higher risk of incidents that disadvantaged skiers at the back of the pack. Overall, 31% of the skiers reported incidents, but none were in R1–10. The overall trend was that lap speed decreased after Lap 1 for all skiers and thereafter remained nearly unchanged for R1–10, while it gradually decreased for the lower-performing groups. Skiers in R31–40, R21–30, and R11–20 lost the leader pack during Lap 3, Lap 4, and Lap 5, respectively, and more than 60% of the time-loss relative to the leader pack occurred in the uphill terrain sections. Ultimately, skiers in R1–10 sprinted for the win during the last 1.2 km, in which 2.4 s separated the top five skiers, and a photo finish differentiated first from second place. Overall, a high correlation emerged between starting position and final rank.

**Conclusions:**

Our results suggest that (a) an adequate starting position, (b) the ability to avoid incidents and disadvantages from the accordion effect, (c) tolerate fluctuations in intensity, and (d) maintain speed throughout the competition, particularly in uphill terrain, as well as (e) having well-developed final sprint abilities, are key factors determining performance during skating-style mass-start cross-country skiing competitions.

## Introduction

Cross-country (XC) skiing is a physiologically and technically demanding endurance sport in which speed, work rate, and energy expenditure fluctuate with constantly changing terrain (1, 2). Moreover, different competition formats in XC skiing vary in distance (i.e., approx. 1.5–50.0 km), style (i.e., classic and/or skating), and type of starting procedure (i.e., individual time trials or mass starts (3). Thus, the corresponding factors of race development (i.e., speed profiles across different terrains, pacing strategies and group dynamics) and their performance-determining impact can also differ considerably (2). Accordingly, understanding race-specific demands and associated performance determinants for each competition format is important for optimising training and race strategies. While individual time-trial competitions in both classic and skating-style are well-described in the literature (4–7) mass-start competitions, which represent the most common competition format, are only briefly examined.

Mass-start competitions in XC skiing were first introduced in the 2002 Olympics (8), and, in the most recent Olympics and World Championships, five out of six races were performed as head-to-head competitions, in which the winner is the first person to cross the finish line (8). In mass-start competitions, all skiers start together, often on narrow tracks with limited possibilities to advance in the field. Accordingly, tactical choices are crucial but may consequently influence physiological and biomechanical demands (8, 9). For example, changing position in a narrow track across fluctuating terrain, which induces rapid changes in work rate, requires both tactical and technical flexibility (8).

In mass-start XC skiing competitions, tactical flexibility may be particularly beneficial not only for advancing within the pack of skiers, but also for avoiding incidents and disadvantages caused by the accordion effect, which is known to occur in traffic and has been described in road cycling (10, 11). Briefly, the accordion effect occurs when competitors in front have to reduce speed but soon after accelerate, then the fluctuation in speed propagates backwards and typically increases further back in the pack (10, 11). Although the accordion effect has not been described in XC skiing, the large pack of skiers in mass-start competitions, combined with narrow tracks and fluctuating terrain, likely creates such an effect.

The influence of other competitors complicates individual pacing strategies more in mass-starts than in individual time-trial competitions (12). In mass-start races in mountain biking (13–15) and running (16), most competitors normally follow the leaders for as long as possible in order to benefit from the drafting effect and thereby improve their chances of winning, as may also be the case in XC skiing. At the same time, in XC skiing time trials, more than 50% of the total time is spent uphill (1, 6), which is the most performance-differentiating terrain (6, 17–19). Even though the influence of different terrain on overall performance in mass-start competitions has not been explored, a recent study investigating physiological responses during a laboratory-simulated mass-start competition revealed that the steepest and longest uphill segments were most performance-differentiating (20).

Against that background, the aim of our study was to investigate speed profiles, pacing strategies, group dynamics and their performance-determining impact in a XC skiing mass-start competition.

## Materials and methods

### Participants and design

The study was conducted in Gjøvik, Norway, on the 29th of January 2022 during a mass-start XC skiing competition in the skating style for senior men in the Norwegian National Cup Series. The skiers were recruited in collaboration with the event organisers after receiving information in the team captains meeting two days before the competition and during the distribution of bibs on the competition day. The 57 highest-ranked skiers (i.e., with the lowest FIS distance points) were equipped with global navigation satellite system (GNSS) sensors during the competition and afterward completed an online questionnaire addressing their strategies and experiences during the competition. Of the 57 skiers recruited, the 42 who finished within top 45 were included in our analyses. However, four of these (i.e., ranks 3, 7, 8, and 26) had low-quality GNSS signals, while three (i.e., ranks 18, 42, and 43) did not wear GNSS sensors as they were not among the 57 highest-ranked skier. Therefore, to include speed-profiles from all 45 skiers, we developed a method to synthesise data regarding position and time along the racecourse for those seven skiers with missing speed profiles; we derived a model using a deep learning approach (i.e., a machine learning) with the official race timing (i.e., 17 points along the racecourse) of the 38 skiers with speed profiles of adequate quality as input data. To group skiers by performance level, the top 40 skiers were divided into four groups based on their final rank in the race: R1–10 for ranks 1–10, R11–20 for ranks 11–20, R21–30 for ranks 21–30, and R31–40 for ranks 31–40. Skiers with synthetic position data were excluded from calculations of speed, while skiers ranked 41–45 were not placed in any performance-based group but were nevertheless included in the study to visualise a more realistic pack dynamic. We defined a *pack* as a group of skiers in which the gap between consecutive skiers is less than 3 s. The skiers' self-reported anthropometrics, along with the performance level of the top 45 skiers (*n = *42) and the four performance-based groups, are presented in Table 1.

**Table 1:** Anthropometrics and performance levels [mean value ± standard deviation (mean limits of confidence)] of the analyzed cross-country skiers in a 21.8 km mass-start competition, both overall and for the different performance-groups.

Variable	All R1–45 (*n = *42)	R1–10 (*n = *10)	R11–20 (*n = *10)	R21–30 (*n = *9)	R31–40 (*n = *10)
Age (years)	24 ± 3 (23,24)	26 ± 3 (24,29)	23 ± 2 (21,24)	24 ± 2 (22,26)	22 ± 2 (21,23)
Body height (cm)	182 ± 6 (168,186)	178 ± 5 (174,181)	182 ± 3 (169,181)	185 ± 5 (181,189)	182 ± 7 (177,188)
Body mass (kg)	75 ± 5 (74,77)	72 ± 3 (70,74)	77 ± 3 (75,79)	78 ± 4 (74,81)	75 ± 5 (71,79)
Body mass index (kg·m^2^)	22.7 ± 1.0 (22.4,23.0)	22.9 ± 0.6 (22.1,23.4)	23.3 ± 1.1 (22.4,24.1)	22.6 ± 0.9 (21.9,23.3)	22.5 ± 0.8 (21.9,23.1)
FIS distance points	47.6 ± 18.3 (41.9, 53.3)	26.1 ± 6.5 (21.5,30.8)	41.5 ± 11.4 (34.5. 53.2)	55.5 ± 12.7 (44.0,62.4)	60.5 ± 15.2 (52.5, 73.5)

R1–10 denotes ranks 1–10, R11–20 ranks 11–20, R21–30 ranks 21–30, and R31–40 ranks 31–40.

### Measurements

The skiers were equipped with 10 Hz GNSS sensors (AdMos, Advanced Sports Instruments, Lausanne, Switzerland), a multisensory device comprising an inertial measurement unit in addition to the GNSS sensor, previously validated in alpine skiing (21). The sensors were placed on the skiers' backs, attached to the inside of the race bibs in customised pockets. To assess the accuracy of the GNSS device in that position, the times from the GNSS measurements were compared with the official split times provided by the organiser, giving a mean offset of less than 0.01 s with standard deviation (SD) 0.30 s over all 17 split times and 42 skiers. Within three weeks (6 ± 5 days) after the competition, the skiers (*n* = 42) completed an online questionnaire gathering self-reported anthropometrical characteristics as well as quantitative and qualitative data concerning planned and actual tactics during the competition, speed profiles, and perceived opportunities and challenges. The first six quantitative items referred to the skiers' strategies prior to the competition, whereas the following 11 referred to their experiences during the competition. For all items, the skiers rated their agreement on a 10-point scale (1 = *I do not agree at all*, 10 = *I agree completely*). Meanwhile, the six qualitative items referred to additional strategies prior to and experiences during the competition. The questionnaire was aligned with the objective sensor data and made by an expert group consisting of experienced coaches and researchers in the field. In addition, pilot tests were performed in front of the competition to assure that the questions were relevant and understandable.

### Data processing

**Figure 1:**
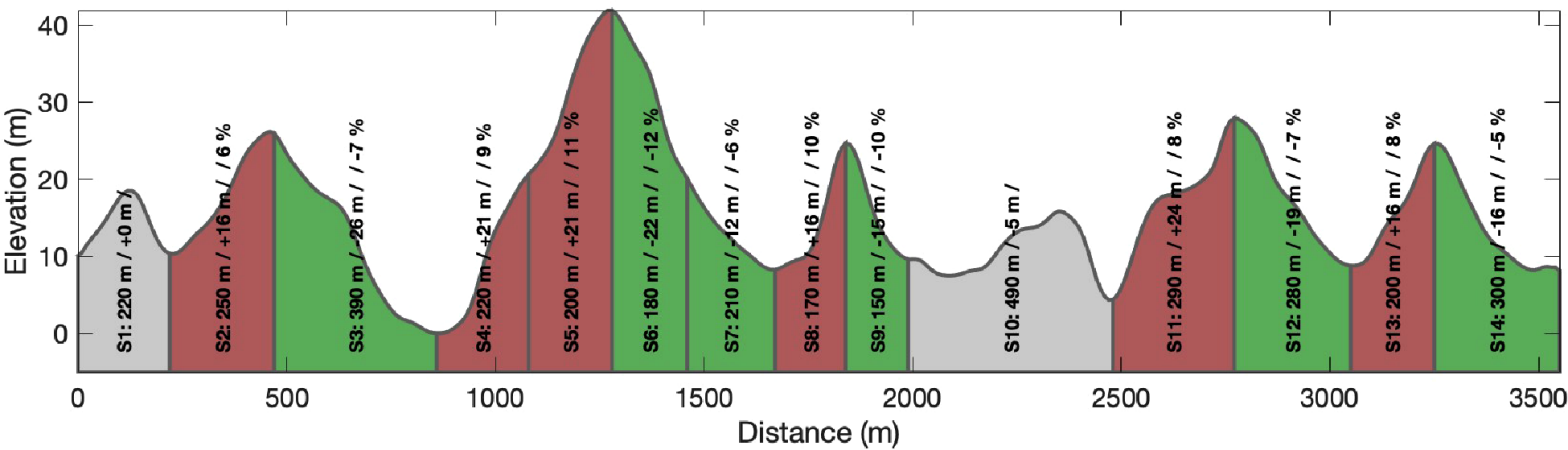
Two-dimensional profile of the racecourse used on Laps 2–6 in a 21.8 km cross-country skiing mass-start competition, showing elevation [m] as a function of lap-distance divided into different terrain segments (S1–S14) with segment distance [m], climb [m], and inclination [%] visualised. Lap 1 was shorter than the other laps but consisted of all segments except S5 and S6. The uphill segments are displayed in red, flat segments in grey, and downhill segments in green.

**Figure 2:**
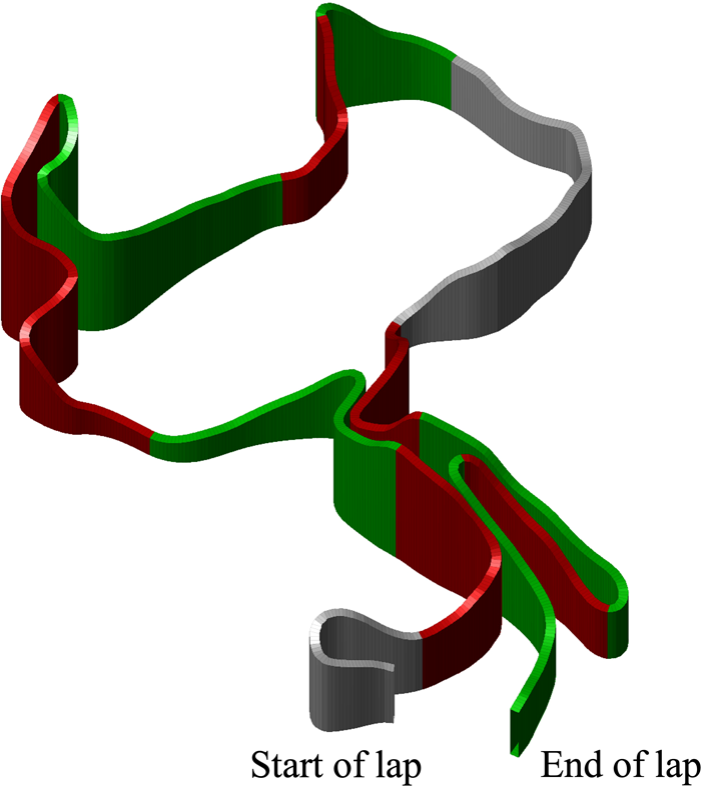
Three-dimensional visualisation of the racecourse used on Laps 2–6 in a 21.8 km cross-country skiing mass-start competition. The uphill segments are displayed in red, flat segments in grey, and downhill segments in green.

The sensor data was processed using MATLAB version R2020a (MathWorks Inc., Natick, MA). A 3D profile of the 21.8 km racecourse was developed based on the GNSS data by averaging data indicating the location and elevation of all skiers during all laps with a resolution of 1 m along the racecourse, after which the individual GNSS tracks were fitted to the racecourse. Segment times were calculated using the time mapped to the racecourse, while segment speed was calculated as course distance divided by time in the respective segments. The racecourse was divided into uphill, flat, and downhill segments based on position and altitude along the course, following a previously described procedure (5). The total uphill, flat, and downhill sections constituted 37.2%, 20.4%, and 42.4% of the total racecourse distance, respectively. To enable lap-to-lap analyses, a 3,550 m long lap course, with a maximal height difference of 42 m and a total climb of 114 m, was defined for Laps 2–6 by excluding the first few metres from the start and finish line. This lap was further divided into 14 segments (S1–S14) based on the type of terrain; Figure 1 shows a 2D elevation profile and Figure 2 a 3D visualisation of the lap course. A separate lap course was developed for Lap 1, which was shorter than the other laps; that lap course was 3,170 m long, with a maximal height difference of 21 m and total climb of 93 m, and, for the skiers’ safety, excluded the steepest downhill segment (S5), one with a sharp curve, and the corresponding uphill (S6). Thus, Lap 1 consisted of 12 of the 14 sections from Laps 2–6.

### Statistical analysis

All continuous measures are presented as mean ± SD. The Shapiro–Wilk test and the visual inspection of histograms were used to assess the normal distribution of the continuous variables. Between-group comparisons for each segment and lap and between-lap comparisons for each segment and group were performed using one-way ANOVA. In cases of statistically significant differences between groups, Tukey's post hoc analysis was conducted for comparison. Correlations between start position and final rank were calculated using Spearman's rank test.

The quantitative data from the questionnaire, reported on a 10-point scale, were presented as median and interquartile range (IQR). Between-group differences for each item were examined using an independent sample Kruskal–Wallis *H* test, and, if statistical differences were found, then pairwise post hoc tests were performed to identify the differences. By contrast, the qualitative data were assessed and presented at the group level. Following a simplified thematic analysis, encoded thematic statements made by three or more skiers in the same group were summarised and are presented among the results.

The level of statistical significance was set at an α-level of .05. All statistical analyses were performed using SPSS version 26 (SPSS Inc., Chicago, IL, United States).

Due to extensive statistical analyses with multiple comparisons, we decided to exclude some of the *p*-values. This was done for readability reasons and none of the excluded values were related to the main findings of this study. The remaining statistical findings are presented as following: Significant differences (*p* < .05) in average lap speed between neighbouring performance-groups are shown with superscript in the speed profile figures for the full lap, for flat, downhill and uphill terrain, and for all of the specific segments. Statistical comparison of average speed between laps for the different performance groups are given in Table 2, while the overall trends for corresponding differences across terrain types and segments are presented in the text. For the quantitative data in the questionnaire, the *p*-values for the between-group comparisons are presented in Table 3, while the significant differences (*p* < .05) between groups using pairwise post hoc tests are visualized using superscript.

**Table 2:** Differences in average speed [m/s] between laps (white cells) with corresponding *p*-values (grey cells) for the different performance groups in a 21.8 km mass-start competition in cross-country skiing.

R1–10	Lap 2	Lap 3	Lap 4	Lap 5	Lap 6	R11–20	Lap 2	Lap 3	Lap 4	Lap 5	Lap 6
**Lap 2**		<.001	.393	<.001	.965	**Lap 2**		.055	.008	<.001	<.001
**Lap 3**	−0.12*		.027	.58	.001	**Lap 3**	−0.11		0.055	<.001	<.001
**Lap 4**	−0.04	0.07		.001	.782	**Lap 4**	−0.14*	−0.03		<.001	<.001
**Lap 5**	−0.15*	−0.03	−0.11*		<.001	**Lap 5**	−0.36*	−0.25*	−0.22*		.962
**Lap 6**	−0.02	0.10*	0.03	0.14*		**Lap 6**	−0.33*	−0.22*	−0.19*	0.03	
**R21–30**	Lap 2	Lap 3	Lap 4	Lap 5	Lap 6	**R31–40**	Lap 2	Lap 3	Lap 4	Lap 5	Lap 6
**Lap 2**		.024	<.001	<.001	<.001	**Lap 2**		<.001	<.001	<.001	<.001
**Lap 3**	−0.18*		<.001	<.001	<.001	**Lap 3**	−0.31*		<.001	<.001	.035
**Lap 4**	−0.47*	−0.29*		.324	1.00	**Lap 4**	−0.52*	−0.21*		.851	.577
**Lap 5**	−0.58*	−0.40*	−0.10		.391	**Lap 5**	−0.56*	−0.25*	−0.05		.110
**Lap 6**	−0.48*	−0.30*	−0.01	0.10		**Lap 6**	−0.44*	−0.13*	0.07	0.12	

R1–10 denotes ranks 1 to 10, R11–20 ranks 11–20, R21–30 ranks 21–30, and R31–40 ranks 31–40.

* Denotes that the values were statistically significant (*p* < .05).

**Table 3:** Median (interquartile range) of the quantitative data in the questionnaire filled out after a 21.8 km mass-start competition in cross-country skiing, both overall (*n =* 42) and within the different performance-groups, including the *p*-values for the between-group comparisons using the Kruskal−Wallis Test (KWT).

How well do you agree with the following statements about your planned strategies for the competition (plan) and execution during the competition (race) on a scale from 1 to 10?		Total	R1–10	R11–20	R21–30	R31–40	KWT
**Open with an individually optimized speed** *Scale: 1 (do not agree) 10 (fully agree)*	Plan	5.0 (5.0)	5.5 (4.5)	7.0 (5.0)^c^	3.0 (3.0)^b,d^	6.5 (3.0)^c^	0.01
	Race	7.0 (4.0)	7.0 (4.5)	8.0 (2.0)	4.0 (4.3)	6.0 (4.3)	0.22
**Open with the leader-pack and try to follow as long as possible** *Scale: 1 (do not agree) 10 (fully agree)*	Plan	10.0 (4.0)	10 (2.5)	10.0 (4.5)	10.0 (1.3)	8.0 (6.5)	0.13
	Race	9.0 (3.0)	9 (2.5)	9.0 (3.0)	10.0 (1.3)	7.5 (6.8)	0.12
**Open at a speed I could sustain throughout the race without “hitting the wall”** *Scale: 1 (do not agree) 10 (fully agree)*	Plan	5.0 (4.0)	3.5 (3.5)	7.0 (4.0)^c^	3.5 (4)^b,d^	5.5 (4.8)^c^	0.03
	Race	6.0 (5.0)	9.0 (5.5)	6.0 (4.5)	3 (5.3)	6.0 (4.8)	0.10
**Open with a faster speed than optimal in order to draft behind other skiers** *Scale: 1 (do not agree) 10 (fully agree)*	Plan	6.0 (5.0)	4.0 (5.0) ^c^	5.0 (4.5)	9.5 (2.5)^a^	5.5 (6.3)	0.02
	Race	7.0 (5.0)	3.0 (5.0)^c^	5.0 (3.0)	8.5 (3)^a^	7.0 (6.3)	0.01
**Ski controlled/conservative in uphills, even when falling behind** *Scale: 1 (do not agree) 10 (fully agree)*	Plan	5.0 (4.0)	7.0 (3.0)	4.0 (5.5)	5.0 (1.8)	4.0 (5.8)	0.20
	Race	6.0 (4.0)	7.0 (4.0)	6.0 (5.0)	7.0 (3.0)	5.0 (4.8)	0.06
**Stay behind other skiers throughout the entire race to save energy, i.e., avoid being at the front** *Scale: 1 (do not agree) 10 (fully agree)*	Plan	8.0 (5.0)	7.0 (4.5)	9.0 (5)	8.0 (4.8)	6.0 (8.3)	0.87
	Race	7.0 (5.0)	8.0 (5.0)	6.0 (5.5)	8.0 (4.8)	7.0 (8.3)	0.79
Question:							
** How fit did you feel today?** *Scale: 1 (very poor) 10 (very good)*	Race	7.0 (2.0)	7.0 (2.0)	7.0 (1.5)	7.0 (2.3)	5.5 (4.3)	0.39
** To what extent were you able to follow the planned strategy?** *Scale: 1 (not at all) 10 (fully)*	Race	6.0 (4.0)	8.0 (1.5)^d^	7.0 (4.5)^d^	5.5 (3.3)^d^	3.5 (2.8)^a^^,b,c^	<0.01
** Did you copy movement patterns of skiers in front of you?** *Scale: 1 (not at all) 1 (fully)*	Race	6.0 (4.0)	5.0 (5.0)	7.0 (3.5)	7.0 (2.3)	5.0 (4.5)	0.41
**How was the glide of your skis compared to other skiers?** *Scale: 1 (very poor) 10 (very good)*	Race	6.0 (4.0)	7.0 (3.5)^d^	7.0 (5.5)^d^	7.5 (3.5)^d^	3.5 (3.5)^a^^,b,c^	0.03
**How satisfied are you with your performance?** *Scale: 1 (not at all) 10 (fully)*	Race	7.0 (4.0)	7.0 (3.5)	7.0 (4.0)	7.0 (2.3)	4.5 (4.0)	0.12

R1–10 denotes ranks 1–10, R11–20 ranks 11–20, R21–30 ranks 21–30, and R31–40 ranks 31–40).

Plan, planned strategy; Race, race experience.

^a^ ^,b,c,d^This value was significantly difference from the corresponding value of R1–10/R11–20/R21–30/R31–40.

## Results

### Start

**Figure 3:**
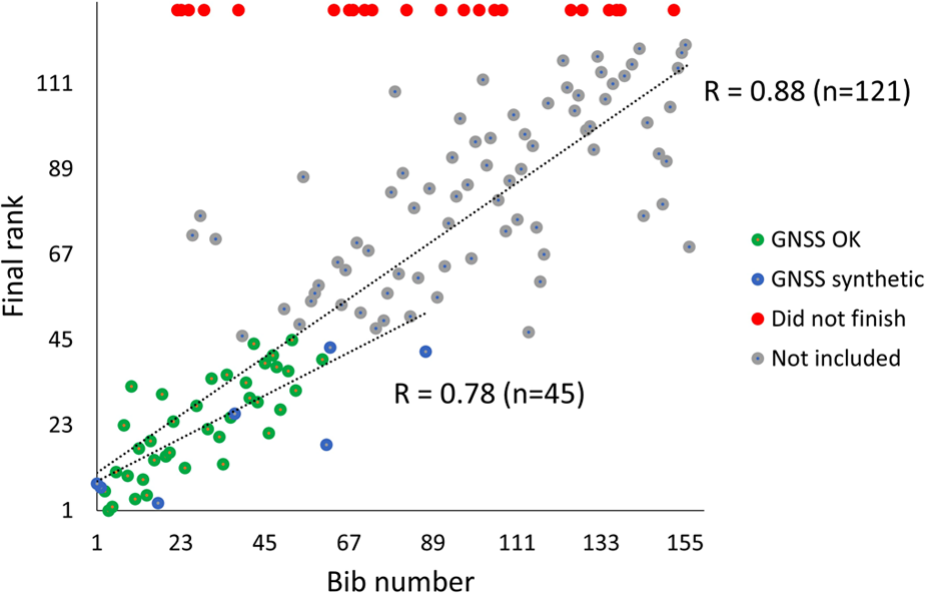
Final rank as a function of starting position (i.e bib number set by FIS distance points) in a 21.8 km cross-country skiing mass-start competition. The included skiers with adequate quality GNSS signals (GNSS OK) are visualised in green, skiers with synthetic speed profiles derived from a deep learning model explained in the methods section (GNSS synthetic) in blue, the skiers who did not finish in red and those not included in grey.

Of the 143 skiers who started the race, 121 finished. Approximately 100 m after the starting line, the time from the front to the end of the field exceeded 16 s. Figure 3 shows the final rank as a function of starting position, with the 45 skiers analysed marked in green (i.e., with GNSS-based speed profiles) and blue (i.e., with synthetic speed profiles). The correlation between the starting position and final rank of the 45 skiers analysed and all 121 skiers who finished the race were *ρ = *.78 and *ρ* *= *.88, respectively (both *p* < .001). The final rank of 80% of the top 45 skiers was within ±15 ranks of their starting position.

### Time behind winner and the formation of dynamic packs

**Figure 4:**
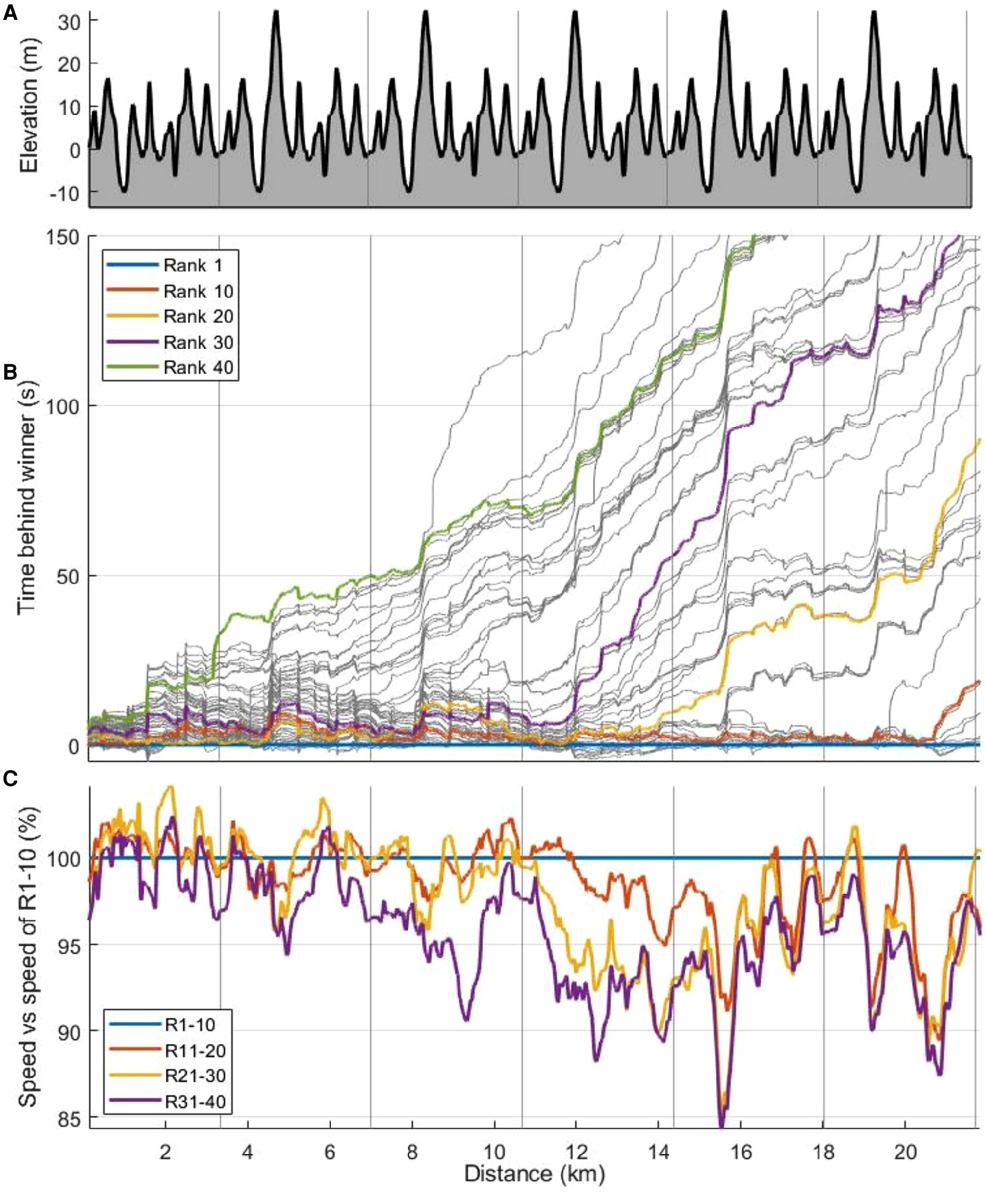
Elevation (**A**), time behind winner [s] for individual skiers (**B**) and continuous speeds (**C**) for the lower performance-groups (group R11–20, R21–30, and R31–40) compared to the first group R1–10 [%] as a function of distance [km] in a 21.8 km mass-start competition. R1–10 denotes ranks 1–10, R11–20 ranks 11–20, R21–30 ranks 21–30, and R31–40 ranks 31–40.

**Figure 5:**
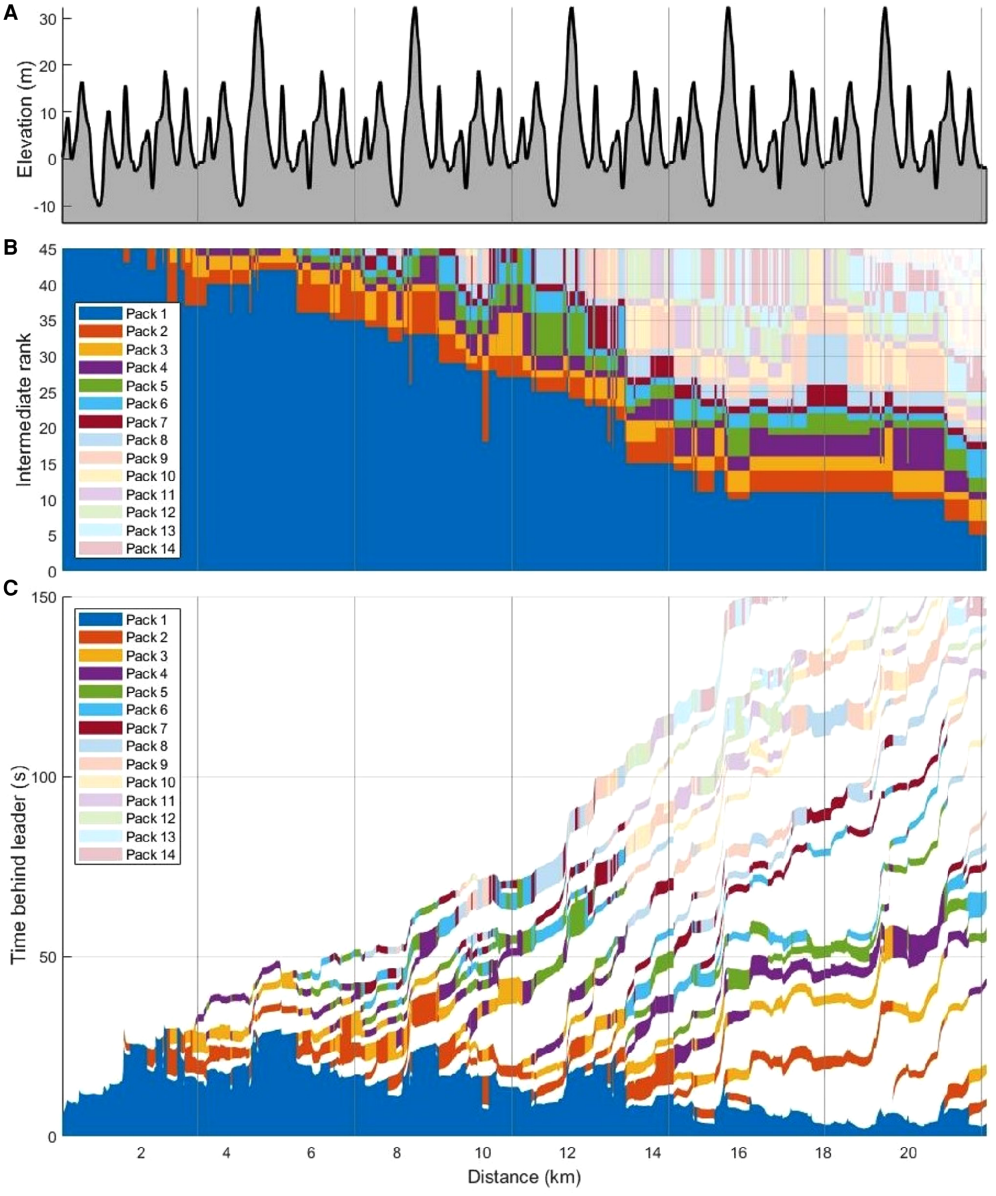
Elevation [m] (**A**), intermediate rank of skiers (**B**), and time [s] behind the current leader for each pack (**C**) as a function of distance [km] in a 21.8 km cross-country skiing mass-start competition. A pack of skiers included all consecutive skiers being less than 3 s apart and each pack is highlighted in different colours.

Individual skiers' times behind the winner, along with continuous speed profiles for the lower-performing groups compared with the best-performing group, are displayed in Figure 4. The figure visualises where the skiers lost time to the winner and shows that all top 45 skiers stayed together in a large pack until 2.3 km, when the pack split into a leader pack and a second pack of skiers who were not able to follow the leader pack. Thereafter, those packs dynamically split and regrouped into smaller packs of two to eight skiers, with some single skiers between packs. This dynamic pack formation, which strongly related to the course's elevation profile (Figure 5A), is visualised as intermediate ranks (Figure 5B) and time behind the current leader (Figure 5C).

### Accordion effect

**Figure 6:**
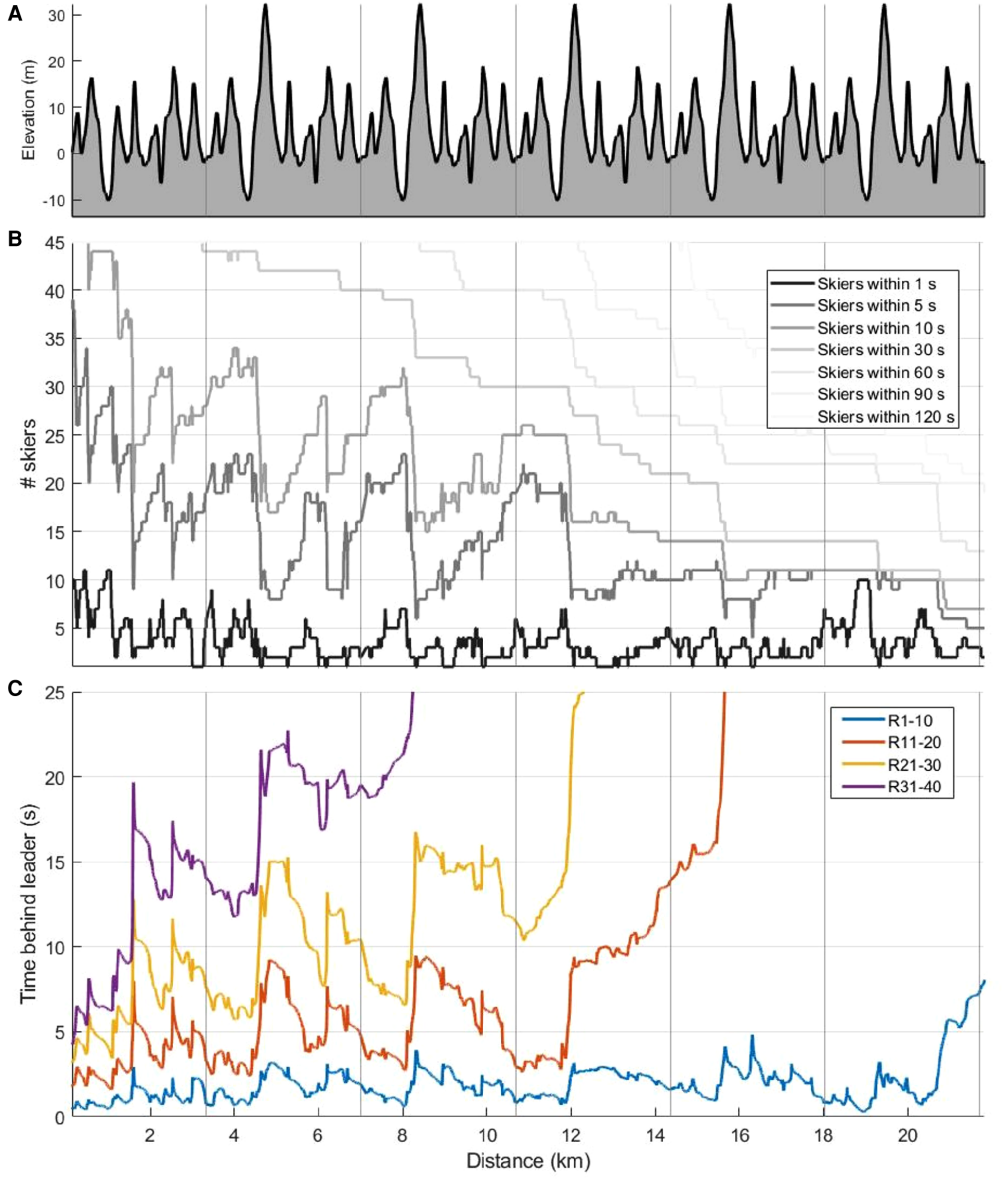
Elevation [m] (**A**), number of skiers within 1, 5, 10, 30, 60, 90 and 120 s from the current leader (**B**) and mean time behind the current leader for the different performance-groups (**C**) as a function of distance [km] in a 21.8 km cross-country skiing mass-start competition. Here, the accordion effect is clearly seen as fluctuating values from 0 to ∼12 km particularly in relation to the longest uphill in for the two lines “skiers within 5 s” and “skiers within 10 s”, and for the three lowest performance-groups (R11–20, R21–30, R31–40). R1–10 denotes ranks 1–10, R11–20 ranks 11–20, R21–30 ranks 21–30, and R31–40 ranks 31–40.

An accordion effect was observed in the first four laps of the competition, particularly in the transition area from downhill or flat terrain to the steepest uphill segments (e.g., from S3 to S4 and from S10 to S11). Figure 6 visualises the effect by showing the number of skiers within 5 or 10 s from the current leader (Figure 6B), as well as the time gap between R1–10 and R11–20, R21–30, and R31–40 (Figure 6C) along the course profile (Figure 6A).

### Pacing profiles

**Figure 7:**
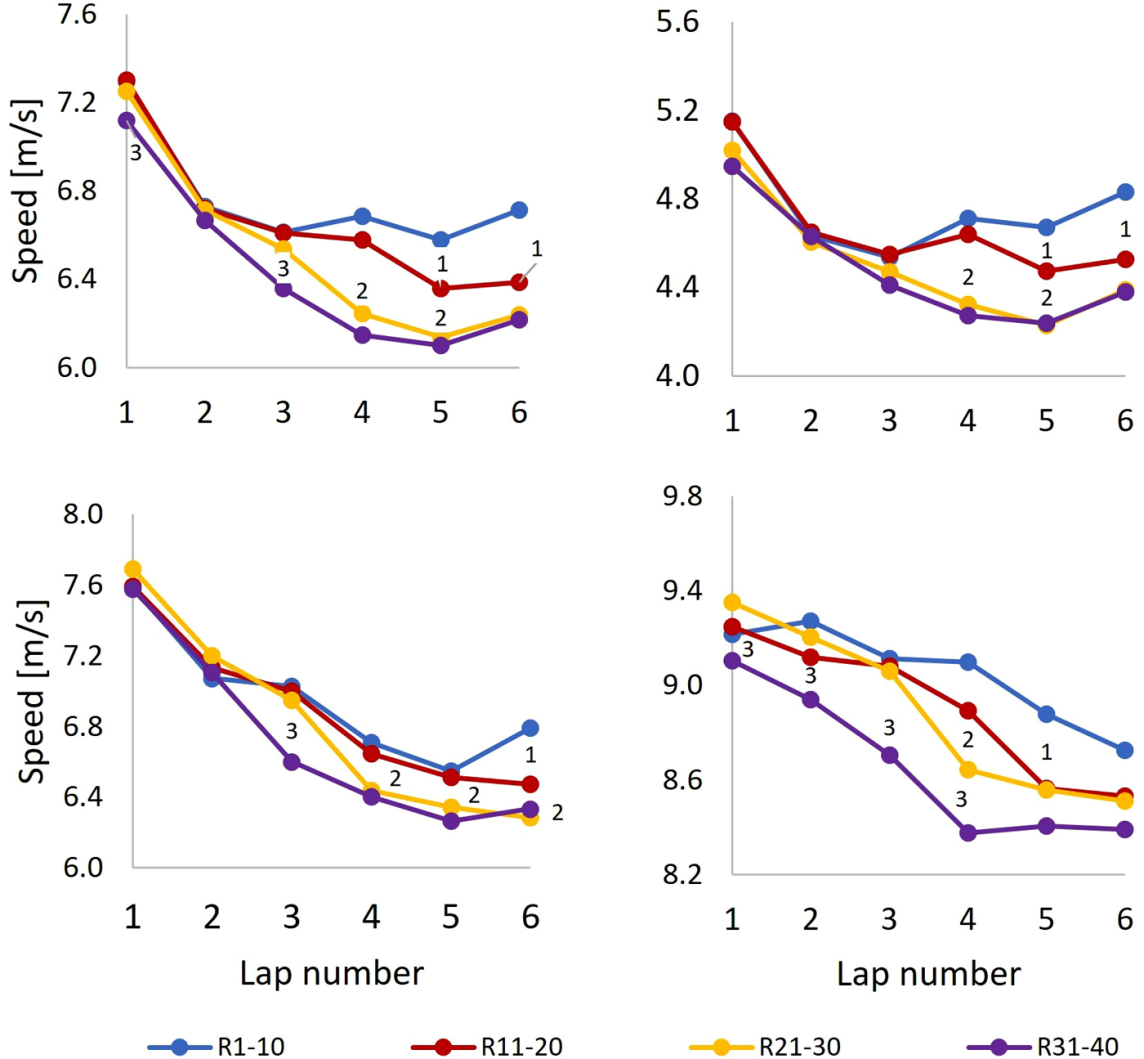
Average speed for full lap, uphill, flat, and downhill terrains as a function of lap-number for the performance-groups during a 21.8 km cross-country skiing mass-start competition. Note that Lap 1 was shorter than the other laps so speed for Lap 1 cannot be compared directly to speed on the following laps. Significant differences in corresponding speed-values between performance-groups are visualised in the figure. R1–10 (group 1) denotes ranks 1–10, R11–20 (group 2) ranks 11–20, R21–30 (group 3) ranks 21–30, and R31–40 (group 4) ranks 31–40. “*N*” on the plots denotes that speed-value for current group was significantly different from group *N*.

Average lap speed for the different performance-based groups with corresponding statistics are shown in Figure 7 “Full lap” and Table 4, respectively. Although speed during Lap 1 could not be compared directly to speed during other laps, the average speed for the parts of the course that could be directly compared across laps (i.e., aggregated average speed for segments S1–S4 and S9–S14), was significantly higher during Lap 1 than during the other laps for all groups (*p* < .001). Later, for R1–10, a relatively even lap speed emerged during Laps 2–6. For R11–20, lap speed was also even during Laps 2–4 but decreased in Laps 5 and 6, whereas R21–30 and R31–40 had reversed J-shaped pacing profiles, with gradually reduced lap speeds from Lap 2 to Lap 4 that evened out in Laps 5 and 6 (statistics given in Table 4). On average, R31–40, R21–30, and R11–20 lost the leader pack during Lap 3, Lap 4, and Lap 5, respectively.

**Table 4:** Summary of encoded statements from the skiers (↑ illustrates the number of skiers) to the open questions in the questionnaire filled out after a 21.8 km mass-start competition in cross-country skiing, divided into different performance-groups.

R1–10	R11–20	R21–30	R31–40
Did you have any planned strategy that was not specifically addressed in the questionnaire?			
↑↑lie far forward in the pack to avoid the accordion effect ↑↑lie behind first part of the race and then try to speed up towards the end ↑overtake in flat /downhill terrain	↑↑lie far forward in the pack to avoid the accordion effect ↑overtake in flat/downhill terrain	↑↑↑ hang on to the leader-pack as long as possible, but not stress with overtakings on the first laps ↑lie far forward in the pack to avoid the accordion effect ↑overtake in flat /downhill terrain	↑↑↑ hang on to the leader-pack as long as possible, but not stress with overtakings on the first laps
What deviations did you do compared to the planned strategy, and why did it not do as planned?			
	↑↑lost time/positions due to an incident ↑↑was not in my best shape ↑bad skis	↑↑↑ lost the leader group earlier than planned ↑↑bad skis	↑↑↑↑↑↑↑↑↑ lost the leader group earlier than planned ↑↑↑↑was not in my best shape ↑↑↑ it was difficult to overtake ↑↑↑lost time/positions due to an incident ↑↑bad skis
Which advantages and/or disadvantages did you experience when skiing closely behind other skiers during the competition?			
Benefits: ↑↑↑↑↑↑ save energy due to less air resistance Disadvantages: ↑accordion effect first part of the race due to uneven speed	Benefits: ↑↑↑↑ save energy due to less air resistance Disadvantages: ↑↑↑ accordion effect first part of race due to incidents, stress, uneven speed, hilltops, coming into uphill segments, and narrow, technical terrain	Benefits: ↑↑↑↑ save energy due to less air resistance Disadvantages: ↑↑↑↑↑↑↑↑ accordion effect first part of race when the pack was large, particularly during two first laps, uneven speed, stress, risk of incidents	Benefits: ↑↑↑↑↑↑↑ save energy because of less air resistance Disadvantages: ↑↑↑↑↑ accordion effect first part of race due to uneven speed, too slow speed into uphill segments, incidents with skier in front
If you copied the movement pattern of skiers in front, which advantages and/or disadvantages did you experience?			
Benefits: ↑ more relaxed if skier in front had similar movement pattern as oneself ↑easier to stay close to the skier in front	Benefits: ↑↑↑↑ more relaxed/easier if skier in front had similar movement pattern as oneself Disadvantages: ↑↑↑ difficult if movement pattern is different to your own	Benefits: ↑↑↑easier to stay close to the skier in front Disadvantages: ↑↑↑ difficult if movement pattern is different to your own	Benefits: ↑↑↑↑↑ reduced risk of incidents in a large pack ↑↑↑easier to stay close to the skier in front Disadvantages: ↑↑↑↑difficult if movement pattern is different to own
Did you have any accidents during the competition?			
	↑↑↑ yes	↑↑↑↑↑ yes	↑↑↑↑ yes
Number of skiers who addressed the accordion effect in the open questions related to two categories, those who a) experienced it or b) strategically tried to avoid it.			
↑↑↑ strategically avoid	↑↑↑ experienced ↑strategically avoid	↑↑↑↑↑↑↑↑ experienced	↑↑↑↑ experienced

R1–10 denotes ranks 1–10, R11–20 ranks 11–20, R21–30 ranks 21–30, and R31–40 ranks 31–40.

Lap speed for the different terrains is shown in Figure 7 which also shows significant differences (*p* < .05) in lap speed between neighbour performance-groups with superscript. The overall trend was that average lap speed decreased lap-to-lap both for flat and downhill terrain but had a similar lap-to-lap variation as the average lap speed for uphill terrain.

**Figure 8:**
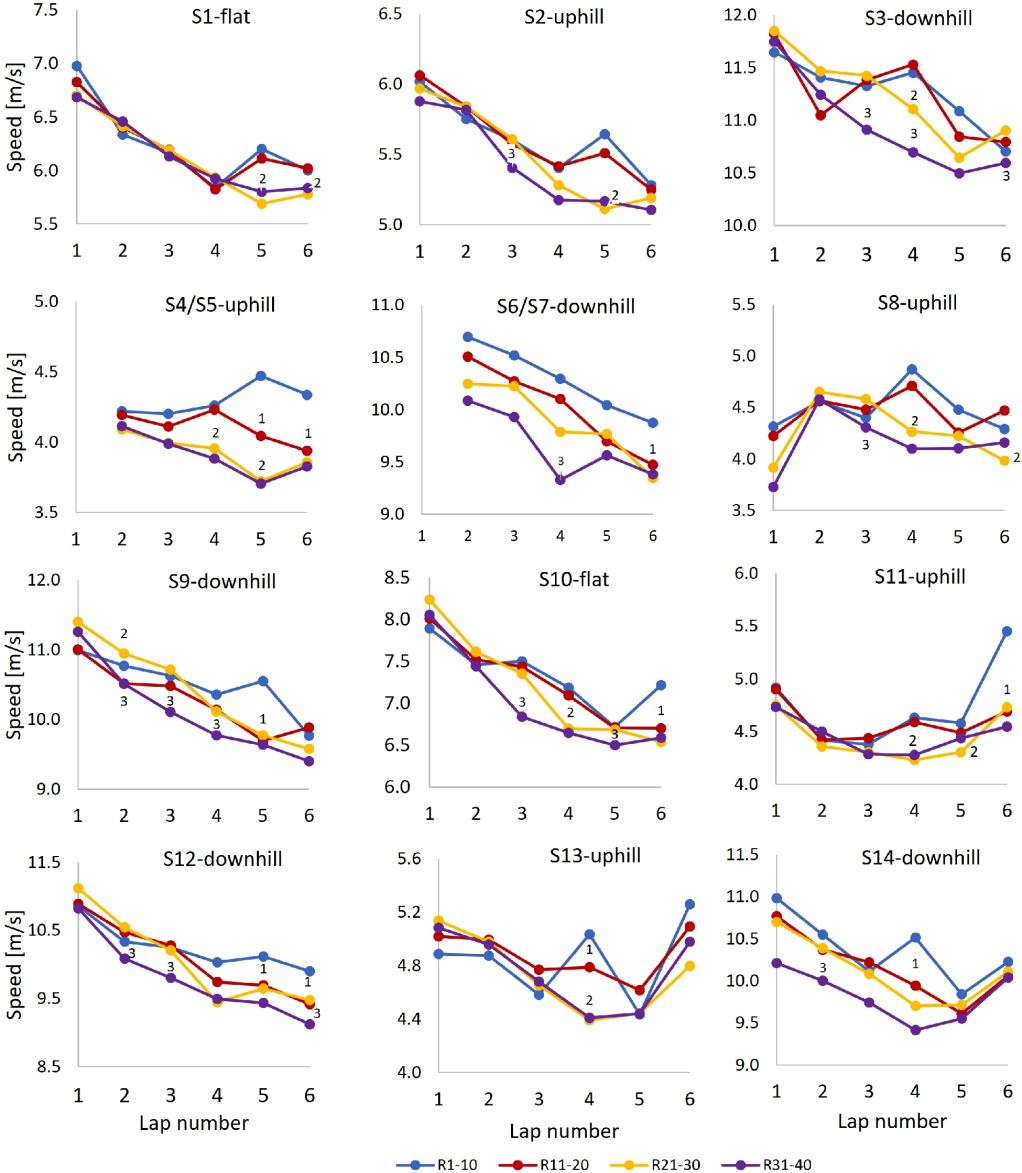
Average speed for each segment (**S**) as a function of lap number for the different performance groups during a 21.8 km cross-country skiing mass-start competition. Note that the speed for S8 on Lap 1 is lower than other laps due to lower speed into this segment since the downhill segment S6 was not included in Lap 1. Significant differences in corresponding speed-values between successive performance-groups are visualised in the figures. R1–10 (group 1) denotes ranks 1–10, R11–20 (group 2) ranks 11–20, R21–30 (group 3) ranks 21–30, and R31–40 (group 4) ranks 31–40. “*N*” on the plots denotes that speed-value for current group was significantly different from group *N*.

Lap speed for each segment is shown in Figure 8. The general trend was that variation in lap-to-lap speed across uphill, downhill, and flat segments was similar to the respective variation in the lap-to-lap speed in the corresponding terrains shown in Figure 7. However, during Laps 4 and 5, the best-performing skiers increased the segment speed in some segments relative to lower-performing skiers, as detailed in Figure 8 where significant differences in corresponding speed-values between neighbour performance-groups are visualised.

### Performance in different terrain

On average, the skiers spent 53.4 ± 0.4% of their overall time in uphill, 19.6 ± 0.2% in downhill, and 27.0 ± 0.3% in flat terrain. The correlations between overall time and time spent in uphill, flat, and downhill terrains were *R = *.97, *R = *.84, and *R = *.87, respectively (all *p* < .001). Compared with R1–10, the relative time loss for R11–20, R21–30, and R31–40 was 61.6%, 74.8%, and 62% going uphill; 11.4%, 11.6%, and 14.2% across flat terrain; and 26.9%, 13.6%, and 23.8% going downhill, all respectively.

### Final sprint

**Figure 9:**
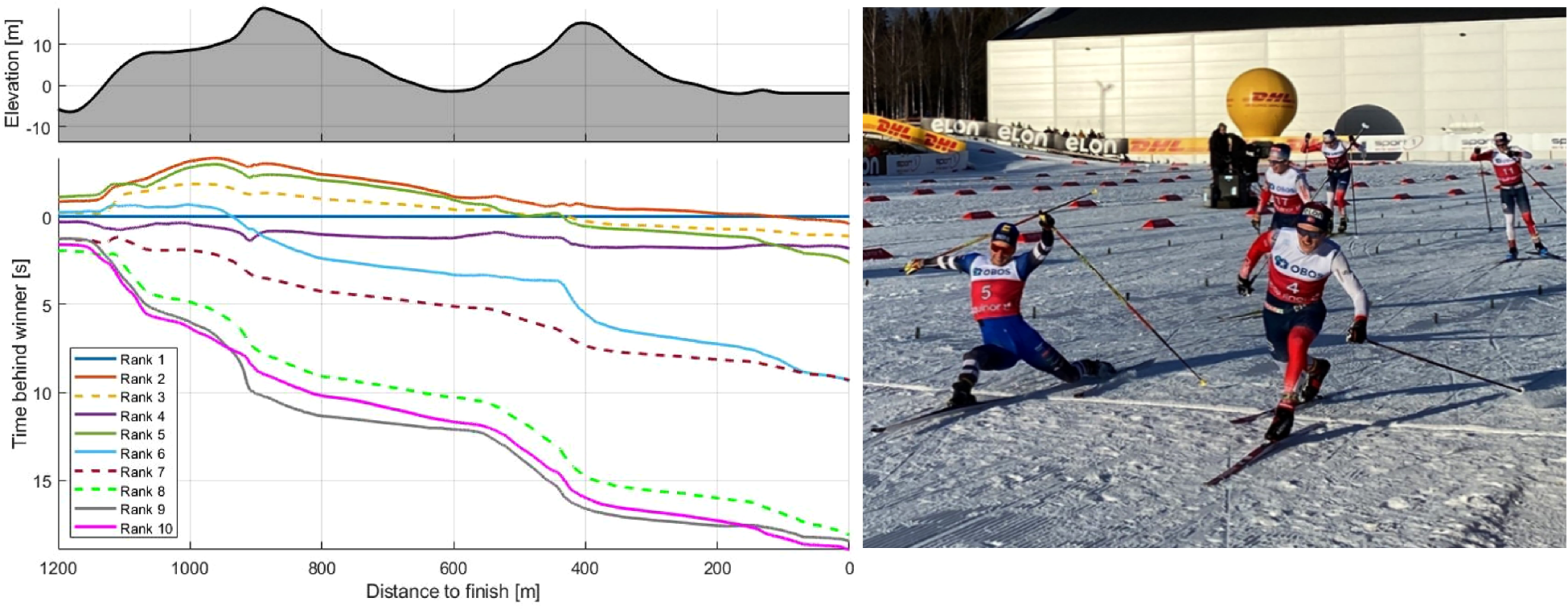
Time behind winner [s] for the top 10 skiers during the last 1.2 km of a 21.8 km mass-start competition (left) and picture of the two best skiers crossing the finish-line (right). Skier with final ranks 3, 7 and 8 have synthetic speed profiles derived from a deep learning model described in detail in the methods.

When approaching the final km, the leader pack consisted of 10 skiers, and the outcome of the competition was decided in a final sprint. All top 10 skiers were within 19 s of each other, the top 5 skiers were within 2.4 s of each other, and a photo finish differentiated first from second place. Figure 9 illustrates each skier's time behind the winner during the final 1.2 km of the race (left) and the first two skiers crossing the finish line (right).

### Questionnaire

Mean responses to the quantitative items on the questionnaire for all skiers and performance-based groups are shown in Table 3. The highest agreement among skiers concerned whether the strategy was to follow the leader for as long as possible even if the speed was too fast [10.0 (4.0) on a 1–10 point scale], with no between-group differences. Significant between-group differences (*p* < .05) emerged for only five of the items: two related to planned strategy, two related to race experience, and one related to both planned strategy and race experience, as detailed in Table 3 shown as blue subscript.

The qualitative statements given by three or more skiers are presented in Table 3. Although the questionnaire did not specifically address the accordion effect, 50% of the skiers mentioned that challenge when responding to the open-ended items. The skiers in R1–10 stated that they had adopted a strategy of staying close to the leader in order to avoid the accordion effect, whereas skiers in the lower-performing groups explained that they had faced disadvantages related to the effect, as shown in Table 3.

## Discussion

In our study, we investigated race development and performance-determining factors in a mass-start XC skiing competition and revealed five major findings. First, all skiers stayed together in a large pack until 2.3 km, at which point lower-performing skiers gradually lost the leader pack and formed new, dynamic packs of two to eight skiers. Second, average lap speed decreased from Lap 1 to Lap 2 and thereafter remained constant among the best-performing skiers, whereas lower-performing skiers gradually decreased their speed throughout the competition, particularly while crossing uphill terrain. Third, a considerable accordion effect occurred for lower-performing skiers during the first half of the competition. Fourth, 10 skiers sprinted for the win during the last 1.2 km, and a photo finish was needed to differentiate first from second place. Fifth and finally, the key factors determining performance were (a) having an adequate starting position (i.e., set by performance level) and (b) the ability to avoid incidents and disadvantages from the accordion effect, (c) tolerate fluctuations in intensity, and (d) maintain speed throughout the competition, particularly in uphill terrain, as well as (e) having well-developed final sprint abilities.

All skiers advanced together in a large pack in the initial 2.3 km of the competition, after which lower-performing skiers gradually lost contact with the leader pack and formed new, dynamic packs of two to eight skiers, with some single skiers between packs. Skiing in large packs is a unique feature of mass-start competitions and facilitates energetic benefits due to reduced ski–snow friction and aerodynamic drag (i.e., drafting) while skiing behind others. The latter is comparable to cycling, in which the aerodynamic drag can be as low as 50% of the drag for an isolated rider at the same speed when moving in a large peloton of cyclists (Blocken et al., 2018). Due to lower speed in XC skiing than in road cycling, the effect of reduced drag is expected to be lower but may play a significant role nonetheless, as demonstrated in classical XC skiing (22). That dynamic also emerged in the questionnaire responses in our study, in which saving energy by reducing aerodynamic drag was reported to be a key motivation for skiing together in packs. Several skiers also reported that it was advantageous to follow the technical patterns of the skier in front of them if they had similar patterns to their own. Even so, that potential advantage was perceived as being stressful if the technical pattern of the preceding skier was different. In terms of aerodynamics, it may be advantageous to synchronise the motion with the skier in front for two reasons. First, as shown in cycling (23) and speed skating (24), a synchronised movement is necessary to achieve a short separation from the skier in front and, in turn, less aerodynamic drag. Second, wind tunnel measurements from speed skating suggest that the reduction in aerodynamic drag is greater if competitors move in synchronised than in unsynchronised movements. Added to that, setting one's skis in the same tracks as the skier in front of them lowers the ski–snow friction for the skier behind. Taken together, the dynamic pack formation observed in our novel analysis of a mass-start XC skiing competition is consistent with what previously has been shown during mass starts in other endurance sport events such as running and triathlons (25, 26).

Moving in large packs may, however, also have disadvantages, particularly for skiers far behind in the pack. Several skiers in our study reported challenges with overtaking other skiers during the competition. Given that difficulty, a starting position in the front of the pack may be crucial for the final rank. However, because the starting position was based on previous performances (i.e., FIS distance points), we do not know how much of the variance can be explained by difficulties in overtaking competitors and thus cannot establish any cause–effect relationship. In a World Cup mass-start race in XC mountain biking, in which the size of the starting field was similar to that in our study (i.e., approx. 100–250 starters) and overtaking other athletes was shown to have similar challenges as in XC skiing, it was found that finishing position depended heavily on starting position (27). Typically, most competitors did not vary in finishing position compared with their starting position by more than ±15 places among elite men and ±10 places among elite women. A similar trend emerged in our study, in which the final rank of 80% of the top 45 skiers was within ±15 places of their starting position. In view of those results, future research should examine the advantages and disadvantages of starting position and whether changes in the starting order or restrictions on course layout are necessary for a fair competition.

The GNSS-based data revealed a considerable accordion effect at the back of the pack during the first half of the competition. Although the accordion effect previously has been described in road cycling (10, 11), our study is the first to reveal it in XC skiing. The effect likely depends on the racecourse, including both the elevation profile and the number and type of turns, along with the number of skiers who start together, the snow conditions, and the skiers' performance level. Our racecourse had several steep, short uphill segments, as well as some difficult sharp turns and many skiers at the same performance level. Thus, there was likely a particularly large accordion effect in the competition, which the skiers described as “large”, “mad” and “extreme”. R1–10 skiers reported adopting a strategy to avoid the accordion effect and showed the success of doing so by remaining at the front of the leader pack. By contrast, skiers in lower-performing groups reported disadvantages such as uneven speed, having too low a speed going into uphill terrain, stressful skiing, and a relatively high risk of accidents, all especially in the first part of the competition and when approaching uphill terrain, crossing hilltops, and navigating narrow, technical terrain. Moreover, those reports are supported by the GNSS-based data. Accordingly, the accordion effect prompted additional decelerations and accelerations for skiers in the back of the pack, which likely had considerable energy costs accompanied by the risk of premature fatigue. Given the obvious disadvantages of the accordion effect, skiers should try to reduce those disadvantages related to the effect during mass-start races. Possible strategies include staying far ahead in the group or, for lower-performing skiers, to leave the leader pack early and ski at their own pace in the first part of the competition in order to have sufficient energy to advance near the end of the competition.

Among other results, several skiers in lower-performing groups reported many incidents and chaotic conditions in the back of the large pack. All told, 31% of the skiers reported being involved in at least one incident during the competition, but none of them were in the highest-performing group (R1–10). Therefore, the ability to avoid incidents seems to be crucial for the XC skier's final position.

Different pacing profiles were observed between the performance-based groups. After a fast start, skiers in the highest-performing group maintained their speed, while lower-performing groups gradually reduced their speed throughout the competition. As revealed by the questionnaire, the skiers had adopted the strategy of following the leader for as long as possible, even if they knew that they could not sustain the pace during all laps, and no between-group differences were found. Adopting that strategy led to positive pacing for lower-performing skiers, who likely had higher relative intensity during the first part of the competition. Such a pacing pattern may be less effective compared to more even pacing strategies shown to be beneficial in individual time trials in XC skiing (Losnegard, 2021). Indeed, that possibility aligns with findings from a laboratory-simulated mass-start competition (Seeberg et al., 2021) in which skiers who fatigued due to high uphill intensity were unable to maintain speed throughout the competition and/or reach their race peak VO_2_/heart rate in the final sprint. The strategy of following the leader for as long as possible has also been observed during mass-start competitions in other endurance sports such as running and triathlon (25, 26) but never before in a mass-start XC skiing competition.

Although lap speed in the leader pack remained fairly constant during Laps 2–6, Figure 8 shows that their speed temporarily increased during some of the segments in the second half of the competition. The leader pack also achieved a higher speed during the last part of Lap 4 and most of Lap 5, after which their speed decreased for a while before increasing again in the final sprint. Such pacing was also commented on in the questionnaire by a skier in R1–10: “Laps 4 and 5 were hard, as expected, but the first part of the last lap was easier. I wasn’t able to keep up when the speed increased again”. Accordingly, the ability to ski at high speed over time and tolerate rapid variations in speed and intensity during the last part of the competition distinguished the highest-performing skiers from their lower-performing peers. That trend aligns with the findings of a track-and-field study in which world-level competition data were examined to identify pacing and tactics across distances ranging from 800 m to 10 km (16). In that study, the medallists were able to not only maintain high speed throughout the entire competition but also accelerate near the end, whereas lower-finishing athletes were able to keep the pace temporarily before slowing down or being unable to accelerate as much as the medallists (16). Therefore, the requirement of tolerating high speed over time in addition to brief fluctuations in intensity is unique for XC skiing compared to other sports and particularly pronounced in mass-start competitions. It may therefore be beneficial to include such features in training sessions—that is, to practice variable intensities during long tempo sessions and develop final-sprint abilities in a fatigued state.

As consistently observed in time trials (6, 17–19) and a simulated mass-start in XC skiing (Seeberg et al., 2021b), uphill terrain was found the most performance-determining in the mass-start competition that we investigated. However, there were also between-group speed-differences in the downhill terrain, in which R31–40 had a constantly lower average speed than all other groups in all laps. Several factors might have contributed to that difference in downhill performance—for instance, more incidents for lower-ranked skiers, less technical and tactical downhill skills, the lack of acceleration over hilltops (28, 29), and the accordion effect. In addition, skiers in R31–40 alone reported having less competitive skis than their peers. In contrast to uphill and downhill terrain, speed along flat terrain was similar in all groups except in the final lap, where R1–10 had higher speed than all other groups in the final sprint. Accordingly, uphill performance, as previously shown in time trials, was also a major determinant of performance in the skating-style mass-start competition that we examined.

The final sprint began 1.2 km before the finish line, when all skiers in R1–10 were together in the leader pack, before the current leader accelerated on a short uphill climb (S11), and three skiers immediately lost contact with the group. In the end, five skiers approached the final 400 metres in such proximity that the outcome of the competition was decided in an all-out-sprint. Ultimately, 2.4 s separated the top five skiers, and a photo finish was needed to differentiate first from second place. Accordingly, many competitors demonstrated a relatively similar performance level, and only marginal time differences distinguished them. Therefore, the ability to generate high speed at crucial moments and in the final sprint is another essential factor of performance in mass-start XC skiing competitions (1, 2, 20).

### Strengths and limitations

The main strength of our study was its exploration of an official FIS-regulated mass-start XC skiing competition with more than 140 participants, including many nationally renowned and world-class skiers. We equipped 57 skiers with high-end GNSS sensors and were able to measure speed profiles for most of them. Although limited snow made the racecourse short and narrow, the temperature, snow conditions, and tracks remained relatively stable during the competition, thereby providing even conditions for all skiers. Another strength of the study was its combination of objective speed profiles with subjective information gained from the questionnaire. A limitation of the study, however, was that some GNSS sensors did not have adequate time in open space prior to the competition due to practical challenges. Therefore, the GNSS signals were poor for a few of the skiers. Additionally, GNSS technology is not accurate enough to detect relative positions in the field, which thereby limited our ability to examine group-based dynamics. A further limitation of the study was that the anthropometric data was self-reported. Also, the questionnaire made for the purpose of the study has not been validated and must be interpreted with caution.

## Conclusion

This study provides the first scientific description of race development and performance determining factors in a mass-start XC skiing competition. All skiers initially clustered together in a large pack, after which weaker skiers gradually fell from the leader pack and formed new, dynamic packs of two to eight skiers throughout the competition. Following a fast start during Lap 1, at a time when skiers positioned themselves, lap speed decreased gradually for all skiers except the ones in the top 10, who achieved relatively constant lap times from Lap 2 and throughout the competition. As expected, performance in uphill terrain was the most pronounced factor differentiating skiers' performance. However, unlike in previous studies on individual time trials, other factors played a role, including a considerable accordion effect during the first half of the competition for the skiers in the back of the pack. Among the top 10 skiers, the final ranks were determined in the last 1.2 km, with a photo finish determining the winner of the competition. The key factors determining performance were (a) having an adequate starting position (i.e., set by performance level) and (b) the ability to avoid incidents and disadvantages from the accordion effect, (c) tolerate fluctuations in intensity, and (d) maintain speed throughout the competition, particularly in uphill terrain, as well as (e) having well-developed final sprint abilities. Thus, though mass-start competitions in XC skiing are determined by many of the same factors as individual time trials, and additionally require tactical flexibility, the ability to tolerate fluctuating intensity variations, and final sprint abilities.

## Data Availability

The original contributions presented in the study are included in the article/Supplementary Material, further inquiries can be directed to the corresponding authors.
